# Qualitative and quantitative comparison of cell-free DNA and cell-free fetal DNA isolation by four (semi-)automated extraction methods: impact in two clinical applications: chimerism quantification and noninvasive prenatal diagnosis

**DOI:** 10.1186/s12967-020-02671-8

**Published:** 2021-01-06

**Authors:** Pascal Pedini, Hajer Graiet, Laurine Laget, Lugdivine Filosa, Jade Chatron, Nicem Cherouat, Jacques Chiaroni, Lucas Hubert, Coralie Frassati, Christophe Picard

**Affiliations:** 1 Department of Histocompatibility, Établissement Français du Sang PACA-Corse, 149 Bd Baille, 13005 Marseille, France; 2 Department of Immunohematology, Établissement Français du Sang PACA-Corse, 149 Bd Baille, 13005 Marseille, France; 3 UMR 7268, ADÉS Aix-Marseille Université/EFS, CNRS, 27 Bd Jean Moulin, 13385 Marseille Cedex 05, France

**Keywords:** cfDNA, Rhesus, Chimerism, Digital PCR, NGS, BIABooster

## Abstract

**Background:**

Non-invasive molecular analysis of cell-free DNA (cfDNA) became a sensitive biomarker for monitoring organ transplantation or for detection of fetal DNA (cffDNA) in noninvasive prenatal test. In this study, we compared the efficiencies of four (semi)-automated cfDNA isolation instruments using their respective isolation kit: MagNA Pure 24 (Roche®), IDEAL (IDSolution®), LABTurbo 24 (Taigen®) and Chemagic 360 (Perkin Elmer®). The cfDNA was isolated from 5 plasma samples and the Rhesus D (RhD)-cffDNA from 5 maternal plasmas. The cfDNA were quantified by digital droplet PCR (ddPCR), BIABooster system and QUBIT fluorometer. The cfDNA fragment size profiles were assessed by BIABooster system. Chimerism were quantified by home-made ddPCR and Devyser NGS kit. RhD-cffDNA in maternal plasma were detected between weeks 14 and 24 of amenorrhea using free DNA Fetal RHD Kit® (Biorad®).

**Results:**

Statistical tests have shown differences in DNA yield depending on the isolation procedure and quantification method used. Magna Pure isolates smaller cfDNA fragment size than other extraction methods (90% ± 9% vs. 74% ± 8%; p = 0.009). Chimerism was only reliable from LABTurbo 24 extractions using the NGS but not with ddPCR whatever extraction methods. RhD-cffDNA were detected by all isolation methods, although IDEAL and LABTurbo 24 systems seemed more efficient.

**Conclusions:**

This comparative study showed a dependency of cfDNA yield depending on isolation procedure and quantification method used. In total, these results suggest that the choice of pre-analytical isolation systems needs to be carefully validated in routine clinical practice.

## Background

Since the studies of P. Mandel and P. Métais in 1948, it has been clearly established that the blood carries a small amount of free circulating nucleic acid from the release of genetic material by the tissues. This cell-free DNA (cfDNA) is in the form of double-stranded DNA with an average size of 150–180 bp corresponding to the winding of DNA around the nucleosome. Its lifespan is less than 2 h, before it is filtered and eliminated from the bloodstream by the spleen, liver and kidneys. Its origin is not yet fully understood but is probably linked to 3 phenomena: apoptosis, necrosis and to a lesser extent, active secretion. In healthy individuals, cfDNA mainly comes from apoptotic cells [1]. The lengths of the DNA fragments in this case are generally around 185–200 bp [2]. Indeed, in cancerous tissues, the size of cfDNA varies a lot, because in addition to apoptosis, necrosis and autophagy are responsible of the death of cancer cells. In this case, the fragments can reach 450–500 bp [3]. The analysis of the cfDNA sizes is performed by electrophoretic methods. Among the most commonly used, the Experion (BIORAD®), the Bioanalyzer and the Tapestation (Agilent®), the QIAxcel (Qiagen®), the LabChip GX Touch (Perkin Elmer®) can be cited. The most sensitive devices detect concentrations lower than the ng/μL [4–6]. Recently, an innovative capillary electrophoresis system, called BIABooster based on μLAS technology, allows simultaneous DNA concentration and separation operations. With this system, the sensitivity of cfDNA detection reach 20 pg/mL [7]. All studies are agree in less quantitative detection of cfDNA in healthy individuals compared to different clinical situations such as strokes and myocardial infarction, intensive muscular exercises, acute renal failure, hepatic cytolysis, trauma, surgery and cancer. Currently, the quantification of cfDNA is performed by spectrophotometric and fluorimetric techniques such as real time quantitative PCR (RQ-PCR), or by more recent and innovative techniques, such as next generation sequencing (NGS) or digital PCR (ddPCR). Today, cfDNA became an important clinical biomarker for prenatal testing, cancer diagnosis and cancer monitoring. Recently, compared to molecular signatures of gene expression predicting organ-specific rejection, quantification of donor cfDNA can be a universal marker for any type of solid organ transplantation. Indeed, the increase or persistence of a high level of donor cfDNA may signify an acute or chronic rejection confirmed by biopsy in kidney, pancreatic, heart and lung transplant patients [8–13]. The donor cfDNA quantification has been performed by RQ-PCR, NGS or ddPCR. However, the relevance of cfDNA in acute or chronic rejection is variable following the studies. The robustness and the reproducibility of cfDNA extractions could be responsible of these differences. A range of commercially kits are today available for cfDNA extraction from plasma, which might influence quantification of cfDNA, the fragment size distribution of cfDNA, and the chimerism detection, i.e. donor cfDNA quantification, under different pathological conditions and cfDNA quantification and qualification methods used.

Another origin of cfDNA is the cell-free fetal DNA (cffDNA) in maternal plasma from the 4th weeks of amenorrhea. The cffDNA is representative of the entire fetal genome [14], that originate from apoptotic placenta cells (trophoblasts) derived from the embryo [15, 16]. It comprises a minor proportion (approximately < 10% in accordance to the week of gestation) of total cell-free DNA (cfDNA) in the plasma of pregnant women [17]. It has been considered a fetal genetic source for the development of reliable non-invasive prenatal test (NIPT). Thus, cffDNA in maternal plasma is used for NIPT for fetal RHD genotyping, Knowledge of the fetal RhD type allows targeted use of antenatal anti-D prophylaxis, avoiding unnecessary treatment of RhD negative women who carry an RhD negative fetus, as these women are at no risk of immunization. In France, since May 2017, this genotyping can be realize from 11 weeks of amenorrhea. A negative result has to be confirmed on a new sample after, at least, 15 days [18].

In this context, we performed a public contract to evaluate different automated instruments, ensuring optimal traceability of samples and cfDNA extraction kit. In response to this tender, 4 automated or semi-automated extraction methods (MagNA Pure 24 (Roche®), IDEAL (IDSolution®), LABTurbo 24 (Taigen®) and Chemagic 360 (Perkin Elmer®) have been tested on 10 samples. QUBIT fluorometer, ddPCR and BIABooster methods have been used to monitor the efficiency and the integrity cfDNA isolation protocols. The chimerism on cfDNA has been quantified using Devyser NGS method and fetal RhD has been detected from maternal plasma by RQ-PCR using free DNA Fetal Kit® RhD (Biorad).

## Results

### Comparison of cfDNA quantification

**Fig. 1:**
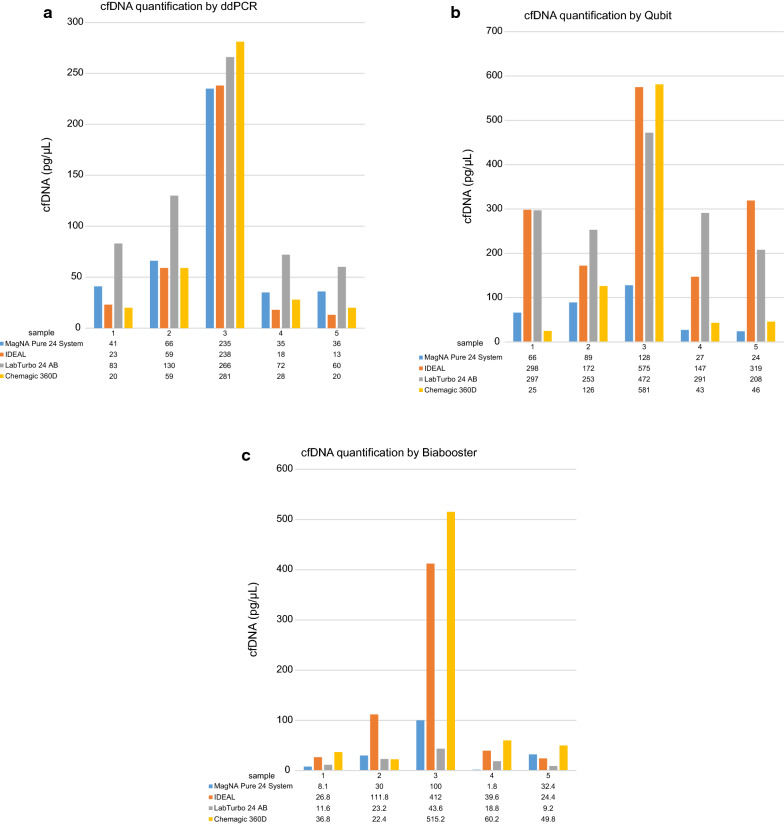
Comparison of the amount of total cfDNA in ng/µL from the 4 cfDNA isolation methods, measured by ddPCR (**a**) by QUBIT HS fluorometer (**b**) and by BIABooster (**c**)

The cfDNA yields obtained with all different isolation methods are summarized in Fig. 1 and Additional file 1: Figure S1. The cfDNA concentrations measured by QUBIT HS were statistically different than those measured by the two other DNA quantification methods (QUBIT HS vs. ddPCR, p = 0.01; QUBIT HS vs. BIABooster, p = 0.01; ddPCR vs. BIABooster, p = 0.78). The values of cfDNA from QUBIT HS revealed quite correlated results with those from ddPCR (rs = 0.5; p = 0.024) whereas the values from BIABooster were not correlated to those from 2 other measuring methods (BIABooster vs. ddPCR, rs = 0.19, p = 0.41; BIABooster vs. QUBIT, rs = 0.269, p = 0.25. When cfDNA concentration was measured by QUBIT HS, the highest cfDNA amount were obtained using the IDEAL and LABTurbo methods compared to the two others methods (p = 0.01). When cfDNA concentration was measured by ddPCR and by BIABooster, the cfDNA amounts were not statistically different for the 4 isolation methods (p = 0.71 and p = 0.43, respectively).

### Comparison of cfDNA integrity

The cfDNA integrity have been evaluated by BIABooster (Tables 1, 2). The size of peak 1 is different depending on the isolation method used (p < 0.005, Table 1). MagNa Pure method gives a peak 1 size significantly different from other extractors (119.75 ± 17.9 vs. 164.5 ± 1.29, p = 0.014). The size of the peak 2 is also different depending on the isolation method (p < 0.0015, Table 2).

**Table 1:** DNA integrity: size of peaks 1 and 2 (A: MagNA Pure 24 System, B: IDEAL, C: LabTurbo 24 AB; D: Chemagic 360D)

Isolation method	A	B	C	D
Peak 1 (pB)	129	165	165	165
	128	163	165	165
	129	166	167	167
	93	164	164	166
	–	164	165	167
Mean (pB)	119.75	164.40	165.20	166.00
SD	17.84	1.14	1.10	1.00
CV	0.15	0.01	0.01	0.01
Min (pB)	93.00	163.00	164.00	165.00
Max (pB)	129.00	166.00	167.00	167.00
*P*	< 0.005			
*p* BCD		0.104		
Peak 2 (pB)	–	307	312	314
	–	301	308	314
	–	312	311	319
	–	309	309	314
	–	305	310	316
Mean (pB)	–	306.80	310.00	315.40
SD	–	4.15	1.58	2.19
CV	–	0.01	0.01	0.01
Min (pB)	–	301.00	308.00	314.00
Max (pB)	–	312.00	312.00	319.00
*p* BCD		0.00158

**Table 2:** DNA integrity: distribution of peaks 1 and 2 (A: MagNA Pure 24 System, B: IDEAL, C: LabTurbo 24 AB; D: Chemagic 360D)

Isolation method	A	B	C	D
75–239 bp				
Mean	90%	79%	73%	71%
SD	9%	7%	9%	8%
CV	10%	9%	13%	12%
Min–Max	79–100%	68–85%	61–84%	62–84%
*P*	0.0789			
*p* B–C–D		0.32		
p240–369 bp				
Mean	5%	15%	15%	15%
SD	5%	3%	3%	3%
CV	99%	18%	19%	18%
Min–Max	0–10%	11–17%	10–17%	11–18%
*P*	< 0.005			
*p* B–C–D		0.865		
370–579 bp				
Mean	1%	3%	6%	7%
SD	2%	3%	2%	3%
CV	148%	114%	40%	47%
Min–Max	0–5%	1–8%	3–9%	2–10%
*P*	0.028			
*p* B–C–D		0.122		
580–1649 bp				
Mean	3%	4%	6%	7%
SD	3%	2%	4%	3%
CV	83%	54%	68%	41%
Min–Max	0–7%	2–7%	2–13%	4–10%
*P*	0.203			
*p* B–C–D		0.314		

Similar proportion of the size profiles of cfDNA was obtained from all isolation methods, except to MagNa Pure method (Table 2). Indeed, analysis of the proportion of size profiles shows that MagNa Pure method is different for smaller size (< 239pB) from the other isolation methods (mean: 90% ± 9% vs. mean: 74% ± 8%, p = 0.009).

### Chimerism quantification

The quantification of the chimerism was performed by ddPCR and NGS (Table 3). The chimerism was not detected by ddPCR, regardless of the isolation method. It should be noted that the cfDNA from the LABTurbo method detect qualitative signals which were quantitatively uninterpretable. Unlike other methods, the LABTurbo method allows a quantification of chimerism using the NGS technique. Eight out of 24 markers were informative. The cfDNA extracted from the LABTurbo method, while respecting the quality validation criteria, determine a chimerism quantification of 2.7% and 16%, consistent with the expected percentages (1 and 10%, respectively).

**Table 3:** Chimerism results of the different isolation methods using NGS and ddPCR techniques

Marker ID	Chromosome	Recipient	Donor	Control gDNA		MagNAPure		IDEAL		LabTurbo24 AB		Chemagic 360	
				100%	15%	10%	1%	10%	1%	10%	1%	10%	1%
NGS chimerism													
1	1	+/−	−/−	100	14.7	16.2**	0.1*	0**	0*	21.8*	4.6	10.6**	5.7*
3	1	+/−	+/+	100	12.8*	3.6**	0*	0**	8.8*	12.7*	2.6	0**	3.3**
8	6	+/−	−/−	100*	18.8*	13.9*	0	0**	2.3*	16.2*	4.9*	12.8**	4*
9	7	+/−	+/+	100*	30.2*	35.8**	0*	0**	2.9*	12.3*	1.9	0**	0.7**
11	8	−/−	+/+	99.1*	18.8*	19.4*	0*	0**	1.5*	10.4*	2.3	5.7**	1.2*
12	9	+/+	−/−	98.1	16.5*	10.6**	0*	0**	3.9*	12.7*	1.2	15**	2.4*
22	20	−/−	+/+	98.9	17.1	7.1**	0**	100**	2.6**	24*	1.8	0**	1.4**
23	22	+/−	+/+	100*	16*	14.8*	0	0**	0*	17.5*	2.8*	8.1**	1*
Average (%)				99.5	18.1	15.2	0	12.5	2.7	16	2.7	6.5	2.4
SD				0.7	5.3	9.8	0	35.4	2.8	4.9	1.3	6.1	1.8
ddPCR chimerism													
Total droplets				22,512	21,496	22,286	22,685	21,481	21,253	16,812	18,665	19,207	19,115
RPP30 positive droplets				13,184	11,256	94	84	40	29	124	128	55	40
Y-marker positive droplets				7121	914	0	0	0	0	4	1	0	0
% chimerism (Y-marker heterozygous × 2)				108%	16%	NC	NC	NC	NC	6%	< DL NC	NC	NC

Quality warning: * (yellow): coverage: 1000–10,000 X, ** (red): coverage < 1000 X

*NC* not calculated, *DL* detection limit

### Detection of *RHD* Cff-DNA

All isolation methods gave the expected results for detection of RHD cff-DNA (Tables 4 and 5). It should be noted that LABTurbo and IDEAL methods had lower Ct values of the exogenous DNA than the two other methods (mean exogenous Ct: 33.71 for IDEAL, 32.86 for LABTurbo vs. 35.5 for MagNa Pure and 37.8 for Chemagic) (Table 4). The Ct values of RHD exon 5, RHD exon 10 and RHD exon 7 differ between them from assay in several samples by having a higher or a lower Ct value.

**Table 4:** Quantification results of exogenous DNA of the different isolation methods using DNA Fetal Kit® RHD CE (BIORAD®)

	MagNA Pure 24 System	IDEAL	LabTurbo 24 AB	Chemagic 360D
Ct exonegous DNA	35.61	33.65	33.08	37.22
	35.61	34.37	32.52	35.64
	36.24	33.79	32.59	38.39
	35.42	33.42	33.25	38.66
	34.66	33.32	32.86	39.38
Ct mean	35.51	33.71	32.86	37.86
SD	0.57	0.41	0.31	1.46
CV	2%	1%	1%	4%
Ct Min	34.66	33.32	32.52	35.64
Ct Max	36.24	34.37	33.25	39.38
*p*	< 0.005

**Table 5:** Quantification results of 3 Rhesus exons of the different isolation methods using DNA Fetal Kit® RHD CE (BIORAD®)

Isolation methods	Origin samples	Weeks	Expected results	Results				
				Ct exon 5	Ct exon 7	Ct exon 10	Ct exonegous DNA	Results
MagNA Pure 24 System	Sample 1	15	Positive	37.12	35.7	38.02	35.61	Positive
	Sample 2	27	Positive	34.24	35.18	35.2	35.61	Positive
	Sample 3	16	Negative	> 40	> 40	> 40	36.24	Negative
	Control D+	ND	Positive	33.18	34.02	34.5	35.42	Positive
	Control D−	ND	Negative	> 40	> 40	> 40	34.66	Negative
IDEAL	Sample 4	24	Negative	> 40	> 40	> 40	33.65	Negative
	Sample 5	20	Positive	38.1	36.88	36.78	34.37	Positive
	Sample 6	14	Positive	36.22	35.15	36.8	33.79	Positive
	Control D+	ND	Positive	32.09	33.12	33.3	33.42	Positive
	Control D−	ND	Negative	> 40	> 40	> 40	33.32	Negative
LabTurbo 24 AB	Sample 7	21	Positive	34.89	36	36.12	33.08	Positive
	Sample 8	19	Negative	> 40	> 40	> 40	32.52	Negative
	Sample 9	14	Positive	37.33	37.18	37.82	32.59	Positive
	Control D+	ND	Positive	32.27	33.08	33.88	33.25	Positive
	Control D-	ND	Negative	> 40	> 40	> 40	32.86	Negative
Chemagic 360D	Sample 10	18	Positive	37.63	37.45	37.87	37.22	Positive
	Sample 11	18	Negative	> 40	> 40	> 40	35.64	Negative
	Sample 12	21	Positive	36.34	38.22	40	38.39	Positive
	Control D+	ND	Positive	35.07	35.44	37.07	38.66	Positive
	Control D−	ND	Negative	> 40	> 40	> 40	39.38	Negative

## Discussion

The cfDNA is today a source of clinical biomarkers in blood sample. However, this utility is limited by its low concentration and small fragment size. The variations of these two characteristics of cfDNA inducing different analytical results depend on the isolation method used. Thus, in response to a tender, this study presents 4 high efficient isolation automats for cfDNA isolation. Three of the four extractors (LABTurbo 24, Chemagic 360, MagNA Pure 24) are CEIVD marked. They are all characterized by an extraction of a maximum of 24 tubes in a time varying from 45 min to 2 h. Two automated systems (LABTurbo 24 and MagNA Pure 24) integrated full process traceability (from the primary tube to the elution).

The IDEAL and LABTurbo methods have a higher extraction yield by QUBIT HS than the two other isolation methods. Other authors who have previously shown that the extractors have different cfDNA extraction yields. Thus, Fleischhacker et al. [18] had compared extraction with Qiagen®, MagNa Pure® and NucleoSpin instruments from 44 samples and showed that the quantification of cfDNA measured by qPCR varied from 1.6 to 28.1 ng/mL depending on the method used. Likewise, Perez-Barrios et al. [19] noted these variations by comparing MagNa Pure® and Maxwell®RSC on 26 samples, using the QUBIT 2.0 Fluorometer and ddPCR. In addition, Sorber et al. [20] also observed variations in cfDNA extraction efficiency using ddPCR, by comparing the QIAamp circulating nucleic acid kit with four other cfDNA isolation kits (the PME free-circulating DNA Extraction Kit, the Maxwell RSC ccfDNA Plasma Kit, the EpiQuick Circulating Cell-Free DNA Isolation Kit, and two consecutive versions of the NEXTprep-Mag cfDNA Isolation Kit).

This study highlights that the 3 assay techniques give different concentrations of cfDNA for the same sample with large variations sometimes, especially between QUBIT and the two other methods. These effects have also been observed by others [21, 22]. An explanation should be the difference in their ability to accurately quantify different fragment sizes of cfDNA. Also, compared with intact (unfragmented) genomic DNA, qPCR measurements showed a 67% reduction in the concentration for DNA fragments with a size of 150 bp, while PicoGreen measurements only showed a 29% reduction [23]. In opposite, the amount of DNA measured by the NanoDrop instrument was not affected by fragmentation to 150 bp, probably because fragmentation does not affect absorbance measurements [24].

The cfDNA amounts measured with the QUBIT fluorometer and the ddPCR are quite comparable, but not with BIABooster. This discrepancy could be explained by RNAse I pretreatment before electrophoretic migration. Indeed, QUBIT HS uses a target-selective dye that emit fluorescence when bound to DNA and amplification dPCR mixes are DNA specific. However, the cfDNA concentrations quantified by ddPCR and QUBIT HS were not modify with RNAse I pretreatment (data not shown).

To the best of our knowledge, both different assays in this study have not yet been included in such a comparative study. Therefore, we cannot state with complete confidence that these different methods are more suitable than the other at quantifying short fragments of cfDNA. Also, these inter-method variations induce a single quantification method for an application in a laboratory. Likewise, they cause difficulties in comparing results between different laboratories, when different DNA concentration measurement methods are used.

Except to MagNa Pure method, other methods give a same cfDNA size profile with a first peak around 165 pB and second peak around 311 pB, the first representing a mean of 76% of cfDNA fragments. The MagNa Pure method isolates a larger small fragment with first peak around 119 pB, representing 90% of cfDNA fragmented. Another study showed that the MagNa Pure® provides a smaller amount of cfDNA with a larger amount of small cfDNA (150–200 bp) measured by Agilent 2100 Bioanalyzer [19]. Kloten V et al. observed the differences obtained during extraction on a silica membrane or on magnetic beads. They noted that if the amounts of cfDNA are relatively similar, the size profile is different depending on the extraction technique used. Indeed, extraction on magnetic beads provides a size profile with a larger amount of small cfDNA fragments (< 600 bp) while extraction on a silica membrane provides a size profile with a larger quantity of fragments of large sizes (> 600 bp) characteristic of cfDNA originating from cells lysed during the extraction process [25]. This ascertainment is not verified in our hand because LABTurbo isolates cfDNA on column extraction and the three others on magnetic beads.

The use of cfDNA in screening for rejection of kidney, heart, lung and pancreatic transplants has been published [8–13]. These studies generally show a persistent increase in donor cfDNA detected in patients with biopsy-confirmed rejection. The donor cfDNA chimerism quantification techniques are either RQ-PCR or ddPCR and more recently NGS, with a sensitivity of each technique around 1%. Our study used the ddPCR and the NGS methods to achieve 10% and 1% chimerism, from male plasma diluted in maternal plasma. Although the results of the patients’ chimerism samples would have been more relevant, the DNA mixture was chosen in order to have sufficient volume of plasma for all extractions and reproducible chimerism results.

Surprisingly, only the LABTurbo method could detect the two percentages of chimerism and only by the NGS technique. An explanation is that this isolation method is one of the two methods that allows the extraction of a large amount of cfDNA when measured by QUBIT HS. Similarly, the difference in results between the two techniques of chimerism quantification can be explained by the ability of NGS to amplify shorter strands (70 bp) than ddPCR (200 bp). In our hand, their sensitivity of chimerism quantification from genomic DNA is similar (i.e. 0.1%; data not shown). However, their sensitivity for the chimerism quantification from cfDNA in an organ transplant or other context has not been compared to this date. Furthermore, the plasma mixes are artificial and these results cannot fully reflect the behavior of cfDNA in a sample.

In context of NIPT, all methods allow the *RHD* cffDNA detection using the Free DNA Fetal Kit® RhD. However, the Ct of the exogenous DNA and the various *RHD* exons are generally lower for the LABTurbo and IDEAL methods than for the others, suggesting a better cfDNA extraction yield for these two methods and inducing probably a higher sensitivity of *RHD* cffDNA detection. The small discrepancy between the Ct values of three *RHD* exons, not exceeding 2 Ct values seems not to depend on isolation methods. It has been suggested than the variation of plasma preparation protocols or the low concentration of cffDNA in mothers’ plasma can induce this difference [26].

Limitations of this study are primarily the small sample numbers, which probably limited the power to observe different effects. The extractions were performed only in two runs, after installation and qualification of the automated system in the laboratory. Also, this study presents assays results without optimization of each isolation method. Each supplier tested only one reagent kit on their instrument; some may offer others. The evaluation of the detection of *RHD*-cffDNA used different plasmas for each isolation method. Likewise, the weeks of amenorrhea of the women sampled are quite different for each method.

Our comparative study was only interested in the applications of clinical interest of our laboratory. However, it suggests in particular that the recent development of new molecular techniques such as digital PCR and NGS facilitates the quantification and qualification study of cfDNA, which is the key to minimally invasive early diagnosis in many clinical applications [27]. In addition to *RHD* fetal blood genotyping, our study must be useful in other prenatal diagnosis such as for sex determination, for detection of fetal aneuploidies including trisomy, sex chromosome aneuploidies, specific microdeletions… [28]. Likewise, our chimerism data could be apply in oncological field for minimal residual tumor detection, metastasis detection, integral tumor profiling in each specific patient, personalized medicine, monitoring of oncological therapy effectiveness and clinical prognosis… [29]. Finally, this study describes different automated and standardized methods of cfDNA extraction, which facilitate the effective use of cfDNA in clinical practice, and not only in research areas [28].

## Conclusion

Finally, 4 (semi-)automated isolation methods have been compared for their extraction efficiency of cfDNA as well as for their fragment size, in a context of chimerism quantification and *RHD* cffDNA detection. Statistical testing showed a dependency of cfDNA yield on isolation procedure and quantification method used. In total, this study suggests that the choice of pre-analytical isolation systems needs to be carefully validated in routine clinical practice.

## Materials and methods

### Biological samples

Six samples are from two healthy subjects (one man and one woman), two artificial mixtures allowing the quantification of a chimerism, a subject with a heart transplantation and a negative extraction control (NEC). The two samples intended for chimerism consist of mixing a “men” plasma in a “woman” plasma at 10% and 1% proportions. Blood was drawn into 10 mL Cell-Free DNA Collection Tubes (Roche Diagnostics®, Mannheim, Germany). Donors and patient provided written informed consent.

For fetal DNA extraction study, the samples are from 20 pregnant RhD negative women, between 14 and 24 weeks of amenorrhea, which the RH fetal status is known. For each method, three fetal RHD positive and 2 fetal RHD negative samples were tested.

### cfDNA isolation methods

Four extraction methods were studied using their respective kit, 3 with the magnetic bead system and 1 with the silica membrane system: for magnetic beads isolation, the MagNA Pure 24 (Roche®) using MagNA Pure 24 Total NA Isolation Kit, the Chemagic 360 using NextPrep-Mag cfDNA isolation kit (Perkin Elmer®) and the IDEAL using IDXTRACT-MAG kit (IDSolution®); for silica membrane system, the LABTurbo using Virus combo kit 24C-LVX480-1000 (Taigen®). The characteristics of each extraction method are described in Table 6. Samples were processed according to the different manufacturers’ protocols.

**Table 6:** Isolation method characteristics

Manufacturer	Roche	ID-Solution	Taigen	Perkin Elmer
Robot	MagNA Pure 24 System	IDEAL	LabTurbo 24 AB	Chemagic 360D
	A	B	C	D
Product	MagNA Pure 24 Total NA Isolation Kit	IDXTRACT-MAG	Virus combo kit 24C-LVX480-1000	NextPrep-Mag cf DNA isolation kit
Type	Bead based	Bead based	Column	Bead based
Sample volume (mL)	2–4	0.5–5	0.3–3	0.5–10
Elution volume (µL)	30–200	30–120 (adaptable)	30–200	30–100
Level of automation	Full	Semi	Full	Semi
Manual pre processing		Reconstitute the PK and incubation (5 min at room temperature), addition of beads + buffers (incubation with vigorous shaking for 30 min at 56 °C). Adaptable distribution of buffers and elution reagents	Wash preparation: addition of EtOH	Add beads, add elution reagent in a bar or tube, reconstitute the Proteinase K
Hands-on-time (min)	0	45	5	20
Automated runtime (min)	90	45	70	70
Total runtime (min)	90	90	75	90
Samples	24	24	24	12
Cost of the instrument	+	+	+	++
Cost of consumables	++	+	++	+
Dimensions (cm)				
Height × width × length	77.5 × 76.5 × 89.9	37 × 57 × 72	87 × 74 × 65	90 × 82 × 90

In all cases, cfDNA was isolated using as starting volume 2 mL of plasma obtained after double centrifugation (1600 g, 10 min at room temperature and 4500 g, 10 min at room temperature) of total blood venipuncture and was eluted in 100 μL, 100 µL, 120 µL and 50 µL, respectively with the supplied manufacturer elution buffer. The isolations were stored at − 20 °C until use. All frozen plasma samples were used in experiments within 1 month of collection.

### cfDNA quantification by digital droplet PCR (ddPCR)

Quantification of cfDNA was performed by ddPCR using the Bio-Rad QX200 System following manufacturer's instructions. The ddPCR reaction mixture was loaded into the emulsification device and droplets were formed by QX200 droplet generator®. The contents were transferred to a 96-well reaction plate and sealed with a pre-heated Eppendorf 96-well heat sealer for 2 s. The cfDNA extracted was amplified separately in a Veriti Thermal Cycler (Applied Biosystems®, Foster City, CA, USA). The non-polymorphic homemade probe assay RPP30 was used for analysis. Briefly, reaction volume is 21 μL using 11 μL of SuperMix [ddPCR ™ Supermix for Probes (No dUTP)], 1 μL of RPP30 mix, 3 μL of water + 6 μL of cDNA [or 6 μL of water for the negative control (NTC)]. The amplification program is: 1 cycle of 10 min at 95 °C, 40 cycles of 15 s at 94 °C and 60 s at 60 °C, 1 cycle of 10 min at 98 °C then cooling to 12 °C.

The chimerism analysis has also been quantified by ddPCR QX200 (BioRad®). The quantification of the chimerism is performed using a specific probe of Y chromosome (SO_2_) and a non-polymorphic endogenous gene (RPP30). Absolute quantities of SO_2_ and RPP30 cfDNA copies are determined using the QuantaSoft software. Briefly, the system uses a 2-color detection system for the wild type (HEX) and mutant (FAM) alleles to count the number of droplets positive for each fluorophore. The ratio of the positive signal in FAM (SO_2_)/positive signal in HEX (RPP30, non-polymorphic gene) illustrates the ratio man/woman.

### cfDNA quantification by fluorometer

All cfDNA were quantified by QUBIT dsDNA HS Assay kit (Thermo Fisher Scientific®, Aalst, Belgium), according to the manufacturer’s data.

### cfDNA qualification and quantification by BIABooster

**Fig. 2:**
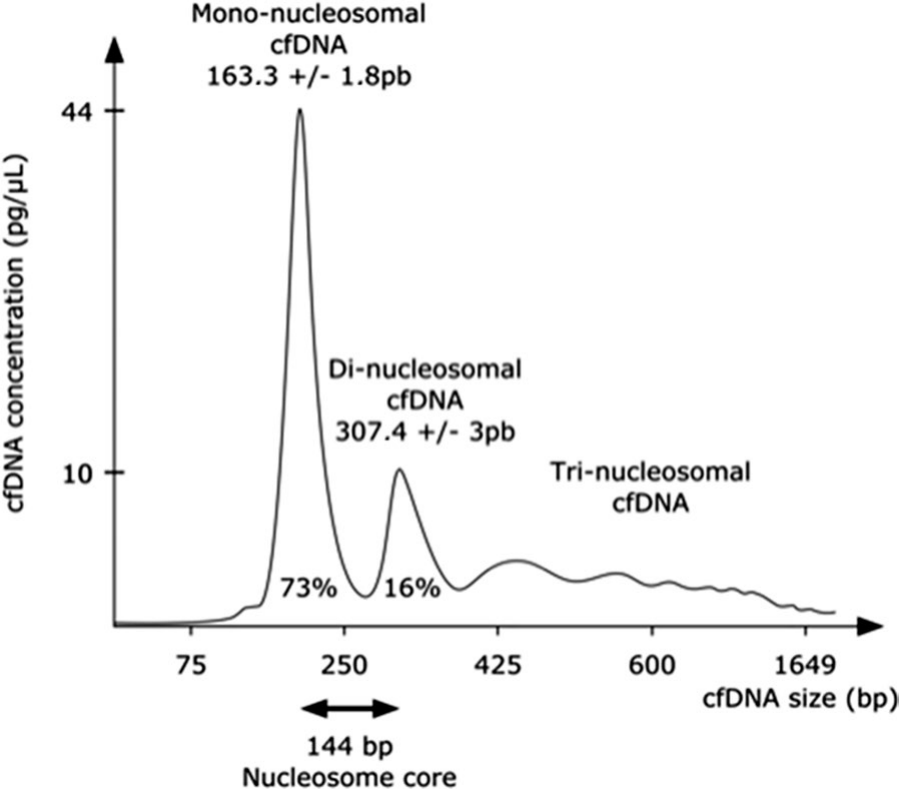
Cell-free DNA size profiles using the BIABooster technique. Capillary electropherogram shows the size of cfDNA isolated from a plasma. The transition from a fluorescence intensity to a concentration is done using the reference ladder. DNA concentration is given for fragment sizes between 75 and 1650 bp. Below and beyond this size range, the information given by the fluorescence intensity constitutes a quantitative indicator of the presence of small and large DNAs respectively, which cannot, to date, be converted exactly in concentration. The relative fluorescence (y-axis) of this ladder is used to calculate the size of the unknown cfDNA samples (x-axis). Thus, any deviation from the baseline, excluding the ladder, indicates the size of cfDNA. Classically, two important size peaks are identified 163 ± 1.8 pB and 307 ± 3 pB. The variations in size of the peaks and in the percentage of each fragment of cfDNA under these peaks come from the study of 120 healthy subjects after cfDNA isolation using IDEAL method (ID-solution®)

Fragment analysis was performed using BIABooster technology (Adelis®). The technology is operated automatically on a commercial capillary electrophoresis instrument using electro-hydrodynamic actuation. All the samples were treated RNase 0.1 U/μL, before analyzing. BIABooster technology enabled analysis of cfDNA fragments between 75 and 1649 bp, according to manufacturer’s protocol. A reference ladder determines the sizes at each pass. Four peaks and six area (< 75 pB, 75–239 pB, 240–369 pB, 370–579 pB, 580–1649 pB, > 1650 pB) are identified (Fig. 2). The cfDNA concentration (pg/µL) is measured under each peak area (Fig. 2).

### Chimerism analysis by NGS

Chimerism level was quantified using Devyser® Chimerism kit. A range of 24 highly informative markers (indels) distributed over 17 chromosomes suitable for screening of a recipient/donor pair and monitoring of chimerism status are used. This kit is based on multiplex PCR followed by analysis with NGS. Only one kit for both screening and monitoring, one tube per sample and one assay for all samples is needed. The protocol is performed in 2 days: the first day for amplification of informative markers, library preparation and quantification and the second for Miseq run and data analysis. Briefly, it is necessary to normalize the DNA to 6 ng/µL before the first PCR (PCR1), then 10 µL of diluted DNA are dispensed into 20 µL of the activated mix. The Thermal cycling of PCR1 is: 95 °C for 15 min, followed by 22 cycles of 97 °C for 30 s, 65 °C for 60 s and 72 °C for 60 s. In a second PCR (PCR2) reaction for library preparation, sequencing adapters including unique index sequences are introduced into each amplicon, enabling pooling of up to 96 samples. Thus, 5 µL of PCR1 diluted to 1/100 (2 µL PCR1 and 198 µL index buffer) are added at 20 µL of Index mix, previously deposited in the wells of the index plate. The thermal cycling of PCR2 is: 95 °C for 15 min, followed by 2 cycles of 97 °C for 30 s, 55 °C for 60 s and 72 °C for 60 s and by 22 cycles of 97 °C for 30 s, 68 °C for 60 s and 72 °C for 60 s. The ramp rates to heating and cooling are 1.6 °C/s. The sample pool (5 µL from each PCR2 well) is purified using the Devyser Library Clean. Purified sample pool is quantified by Qubit kit and sequenced using NGS Illumina chemistry (Micro or Nanoflow cell V2 300 cycles depending of number of sample tested). There is a calculation tool specially designed for Devyser Chimerism sequence planning allowing to optimize the use of the flowcell by calculating the number of genotyping and monitoring possible in a same assay. The resulting sequences are analyzed using the ADVYSER for Chimerism software 1.3 version. Genotyping is determined by the %VAF (variant allele frequency) calculation for each marker using the number of reads classified as “reference” (Ref) and “alternative” (Alt) of the reference sequence hg19 (human genome 19, Genome Reference Consortium). The %VAF is calculated as: Ref reads/(Ref reads + Alt reads). Pre-transplant samples are expected to have a VAF close to 0% (−/−), 50% (+/−) or 100% (+/+). A marker is informative if recipient and donor are homozygous for opposite genotypes or if recipient is heterozygous and donor is homozygous (+/+ vs. −/− or −/− vs. +/+ and +/− vs. +/+ or −/−, respectively). All informative markers are automatically selected but user can deselected those manually. Three warnings should be considered for the selection of the markers: “low coverage” when the minimum recommended coverage of 100 reads/marker is not met, “unexpected VAF” when the %VAF is between 1 and 40% or between 60 and 99% (due to import mix-up (i.e., a post-transplant sample is imported instead of a pre-transplant sample), sample impurity (i.e., can be seen in saliva swabs) or the fact that the patient has had previous transplantations) and “Background noise” when the VAF is between 0.1 and 1% or between 99 and 99.9% (consequence of non-optimal run, index-hopping or carry-over events). Regarding monitoring, ADVYSER performs automatic calculation of % for chimerism level and generation of trend graphs to evaluate monitoring results. For the monitoring, there are two quality criteria (coverage and noise) and for each, there are two levels of warning (“yellow” and “red”). The optimal coverage depends of the % chimerism. Coverage > 10,000 reads is required to call chimerism at 0.1% with high precision and sensitivity. If detected % chimerism is higher (> 1%), a coverage of > 1000 reads/marker is sufficient to determine the % chimerism with high precision and sensitivity.

### Detection of fetal *RHD* in pregnant women

The *RHD* cff-DNA was detected in maternal plasma using the free DNA Fetal Kit® RhD (BioRad®, Hercules, CA, USA), according to the manufacturer’s recommendation. Positive, negative, blank, and extraction controls [exogenous DNA (maize)] are performed in parallel. The presence of the fetal *RHD* gene in maternal plasma DNA is detected by real-time PCR [LightCycler® 480 apparatus (Roche Molecular Biochemicals®, Meylan, France)], targeting three exons: 5, 7 and 10 of the *RHD* gene in a final reaction volume of 20 μL. Results are considered acceptable only if no amplification curve is observed for the negative and blank controls; if the cycle threshold values (Ct) of exons 5, 7 and 10 for the positive control is below 39 cycles; and if the exogenous DNA (maize) is correctly amplified (Ct value < 37 cycles) during the assay. Fetuses were classified as *RHD* positive or negative according to the following result interpretation: the absence of amplification (Ct null, or > 40) for the three exons implies a negative *RHD* sample, and a Ct value between 35 and 40 cycles for two or three exons identify samples as *RHD* positive.

### Statistical analysis

In order to evaluate the amount of the cfDNA levels, the overall mean as well as the coefficient of variation (CV) are determined. The CV is the standard deviation compared to the values mean. cfDNA integrity of the cfDNA isolation kits was established by the comparison of the size of peaks [first (75–239 pB) and second peak (240–538 pB)] and the cfDNA proportion and concentration were calculated under area of these different peaks. The 1-way ANOVA Kruskal–Wallis test was used to compare cfDNA concentrations between distinct conditions. Correlation analysis was performed by calculating a Spearman correlation coefficient. Differences were considered statistically significant if the two-sided *p* were equal or below 5% (≤ 0.05).

## Supplementary Information

**Additional file 1: Figure S1.** Concentration of total cfDNA in ng/µL measured by QUBIT HS fluorometer, by ddPCR and by BIABooster, obtained by the 4 cfDNA isolation methods from 5 samples.

## Data Availability

The datasets used and/or analysed during the current study are available from the corresponding author on reasonable request.

## Abbreviations

cfDNA: Cell-free DNA
cffDNA: Cell-free fetal DNA
NIPT: Non-invasive prenatal test
NGS: Next Generation Sequencing
ddPCR: Digital PCR
RT-PCR: Real-Time PCR
STR: Short Tandem Repeat
RhD: Rhesus D
