# Supplementary material for: Qualitative and quantitative comparison of cell-free DNA and cell-free fetal DNA isolation by four (semi-)automated extraction methods: impact in two clinical applications: chimerism quantification and noninvasive prenatal diagnosis
Source: J Transl Med. 2021 Jan 6;19:15. doi: 10.1186/s12967-020-02671-8 (PMC7788686; doi:10.1186/s12967-020-02671-8)
Supplement: Supplementary file 1 — Additional file 1: Figure S1. Concentration of total cfDNA in ng/µL measured by QUBIT HS fluorometer, by ddPCR and by BIABooster, obtained by the 4 cfDNA isolation methods from 5 samples. [file 12967_2020_2671_MOESM1_ESM.pptx]

## Slide 1
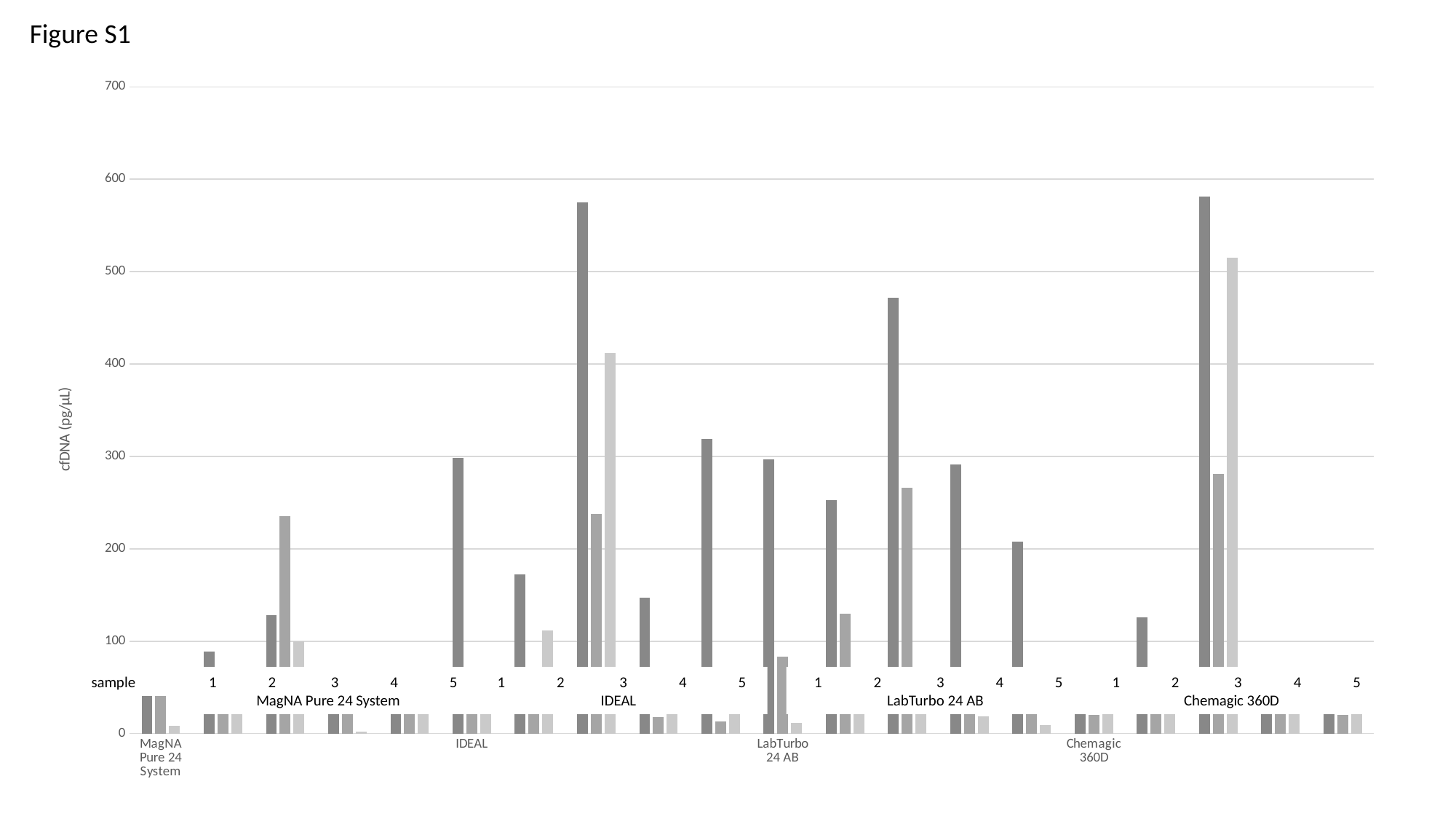

Figure S1
### Chart
| Category | Qubit (pg/µL) | ddPCR (pg/µL) | Biabooster (pg/µL) |
|---|---|---|---|
| MagNA Pure 24 System | 66.0 | 41.0 | 8.1 |
| | 89.0 | 66.0 | 30.0 |
| | 128.0 | 235.0 | 100.0 |
| | 27.0 | 35.0 | 1.8 |
| | 24.0 | 36.0 | 32.4 |
| IDEAL | 298.0 | 23.0 | 26.8 |
| | 172.0 | 59.0 | 111.8 |
| | 575.0 | 238.0 | 412.0 |
| | 147.0 | 18.0 | 39.6 |
| | 319.0 | 13.0 | 24.4 |
| LabTurbo 24 AB | 297.0 | 83.0 | 11.6 |
| | 253.0 | 130.0 | 23.2 |
| | 472.0 | 266.0 | 43.6 |
| | 291.0 | 72.0 | 18.8 |
| | 208.0 | 60.0 | 9.200000000000001 |
| Chemagic 360D | 25.0 | 20.0 | 36.80000000000001 |sample
 1 2 3 4 5
MagNA Pure 24 System
 1 2 3 4 5
IDEAL
 1 2 3 4 5
LabTurbo 24 AB
 1 2 3 4 5
Chemagic 360D
